# Shining a spotlight on females in ADHD science

**DOI:** 10.1002/jcv2.70060

**Published:** 2025-10-28

**Authors:** Stephen P. Becker

**Affiliations:** ^1^ Division of Behavioral Medicine and Clinical Psychology Cincinnati Children's Hospital Medical Center and Department of Pediatrics University of Cincinnati College of Medicine Cincinnati Ohio USA

**Keywords:** attention‐deficit/hyperactivity disorder, developmental psychopathology, girls, sex differences

## Abstract

As *JCPP Advances* marks its fifth anniversary, this editorial highlights the need for continued and expanded research on females with attention‐deficit/hyperactivity disorder (ADHD). Despite growing recognition of sex differences in ADHD presentation, diagnosis, and outcomes, females remain underrepresented in both research and clinical contexts. Drawing on several studies published in this issue, this editorial synthesizes important findings regarding ADHD symptom severity, ADHD‐related impact, and associations with emotional difficulties in females with ADHD. Additional adequately‐powered and developmentally informed studies are needed to elucidate mechanisms, moderators, and resilience promoting factors in females with ADHD across the lifespan. We look forward to seeing more research on this and other pressing topics facing child and adolescent mental health within the pages of *JCPP Advances* as we look forward to the next 5 years.

## Key points

- Females remain underrepresented in attention‐deficit/hyperactivity disorder (ADHD) research and clinical trials, despite growing recognition of sex differences in ADHD presentation and outcomes.
- Studies in this issue find that ADHD's functional impact may be less recognized in girls, females may face a higher diagnostic threshold, and they experience greater emotional difficulties and schoolwork pressure.
- The field needs large, developmentally informed, and adequately powered studies to explore mechanisms, moderators, and resilience factors specific to females with ADHD.
- JCPP Advances calls for continued collaboration and community engagement to expand rigorous, inclusive ADHD research that integrates both risk and resilience frameworks.

With this December 2025 issue, we wrap up the first 5 years of *JCPP Advances*. By all accounts, including the number and quality of submissions, growing readership, and academic indexing and metrics, *JCPP Advances* has been successful in advancing and disseminating high‐quality research in developmental psychopathology and youth mental health. Importantly and impressively, the articles in the first 5 years of *JCPP Advances* have been wide‐ranging in their foci and methodologies, encompassing experimental approaches and clinical trials, theoretically grounded tests of developmental effects and processes, and timely commentaries on important issues facing our field. The journal's first Special Issues have focused on evidence synthesis studies (Vol. 3, Issue 3; Bellato et al., 2023) and participatory research in child psychology and psychiatry (Vol. 4, Issue 4; MacLeod et al., 2024), with transdiagnostic approaches and other Special Issue topics forthcoming.

Each issue assembles outstanding open‐access papers that together advance and shape the field, and the 14 papers in the current issue are no exception. Among the papers in this issue, several underscore the continued importance of research focused on females in youth and young adults with attention‐deficit/hyperactivity disorder (ADHD).

Historically neglected in both research and clinical trials, calls to advance understanding and treatment of girls and females with ADHD are not new. Stephen Hinshaw, a pioneer in this area, recently led an Annual Research Review published in our sister journal, the *Journal of Child Psychology and Psychiatry*, which detailed the substantial underrepresentation, extant research findings, and important research and clinical directions for females with ADHD (Hinshaw et al., 2022). Although major progress has been made, far more remains to be done. Indeed, an international Delphi study published earlier this year found the life course development, assessment, and treatment of females to be among the most pressing research priorities currently facing the field of ADHD (Stephens et al., 2025). Towards this goal, several findings from papers in the current issue add to the existing research literature and shed light on important sex differences in ADHD and the need for more research attention focused on females with ADHD and other neurodevelopmental conditions.

In a UK birth cohort of over 12,000 youth at ages 8 and 11, Horstmann et al. (2025) examined the extent to which ADHD and other traits are associated with ADHD‐related impact in the general population. ADHD traits were associated with significantly higher ADHD‐related impact across both parent and teacher informants in both childhood (age 8) and early adolescence (age 11). However, important sex differences emerged based on both informant and age wherein parent‐reported ADHD traits were more strongly associated with ADHD‐related impact for males than females. The same interaction effect was also found when examining separate inattention and hyperactivity‐impulsivity dimensions. Importantly, all sex differences were more marked at age 11 than age 8. As noted by the authors, sex differences in the link between ADHD traits and their impact on functioning may be context dependent. The moderation findings reported in Horstmann et al. suggest that parents may be less likely to see the functional impact of ADHD traits in females than males, a gap that may widen during the critical transition from childhood to adolescence. Findings from this study have important implications, particularly as parents are generally responsible for pursuing evaluation and treatment for suspected ADHD even when behaviors and their impact are observed by teachers in the school context.

Solberg et al. (2025) directly examined sex differences in self‐reported ADHD symptoms in three non‐overlapping Norwegian cohorts of adolescents and adults, including a clinical cohort and two population‐based cohorts. Unsurprisingly, across all three cohorts, participants with a self‐reported ADHD diagnosis reported greater ADHD symptoms using the 6‐item screening version of the Adult ADHD Self‐Report Scale (ASRS). Perhaps less expected were the sex differences which were also consistent across samples: symptom score differences between individuals with and without ADHD were larger for females than males. This difference was statistically significant in two of the three cohorts, with the largest difference found in the clinical cohort. Considering these findings together, the authors conclude that there may be a higher threshold for females than males to obtain a diagnosis of ADHD. This is an important finding that warrants further investigation, including in studies that can also consider age of onset, functional impairment, and possible co‐occurring conditions involved in referral patterns and diagnostic assessment procedures.

Both ADHD and autism were considered in the study by Capp et al. (2024). In a sample of over 550 young adult twins (ages 20–25 years) classified with high or low ADHD and/or autistic traits, females consistently reported significantly higher levels of depression and anxiety and were also more likely to meet criteria for these internalizing disorders. There was also an interaction effect present between autistic traits and sex for depression and anxiety scores, wherein the association between autistic traits and internalizing symptoms was stronger for females than males. The same interaction effect was not present for ADHD traits and sex. Still, there was a consistent main effect showing ADHD traits to be associated with higher anxiety and depression whether examined dimensionally or categorically. It would be valuable for future research to test whether a similar pattern of findings is found in clinical samples, and also in prospective longitudinal studies which can clarify if patterns across sensitive developmental transitions such as childhood to adolescence and adolescence to adulthood. Nevertheless, the findings in Capp et al. underscore the importance of studies that carefully examine sex differences across different (and co‐occurring) neurodivergent traits.

We now turn to the study by Armitage et al. (2025) which differs from the previous research in that it does not focus on ADHD. Rather, in nationally representative samples of secondary school students (ages 11–16 years) in Wales, repeat cross‐sectional data from 2002 through 2021 were examined for trends in the percentage of students reporting perceived schoolwork pressure and emotional problems. Following a decline in perceived schoolwork pressure from 2004 until 2009, rates then climbed upward by 2021. Interaction analyses demonstrated that increases in perceived school pressure were higher for females than males. By 2021, the percentage of female students who reported experiencing “a lot” of schoolwork pressure (33.5%) was almost twice that of male students (18.6%). Greater perceived schoolwork pressure was associated with higher emotional problems in every survey year and for all students, though once again the increase in emotional problems between 2009 and 2021 was more marked in female than male students. The authors correctly caution that further longitudinal research is needed to establish temporal or predictive associations. They also ground their findings in a larger body of research suggesting that school pressures may be increasingly intensified in females who are also navigating distinct social and sex‐based norms and stressors.

So where do these four studies leave us in advancing our understanding of females with ADHD? It is possible that parents are less likely to see the negative impact of ADHD‐related traits in girls compared to boys (Horstmann et al., 2025), which may contribute to delayed evaluation or potentially no evaluation at all. When referred for evaluation, females may then have to display a higher threshold of ADHD symptoms compared to their male counterparts (Solberg et al., 2025), resulting in potential misdiagnosis or barriers to appropriate intervention. At the same time, adolescent females perceive greater schoolwork pressure than adolescent males (Armitage et al., 2025) which may be especially the case for females with ADHD given the academic challenges and impairments that youth with ADHD frequently encounter. It is possible that these schoolwork pressures and other challenges contribute to the onset or worsening of emotional problems (Armitage et al., 2025), such that by young adulthood females with ADHD are especially prone to higher levels of anxiety and depression (Capp et al., 2024).

Of course, this developmental progression is speculative in the absence of direct data, but these are the types of possibilities the articles in this issue point to as critical next steps. Layered within these findings are other pressing questions, such as the distinction between masking and coping or compensatory strategies, particularly when considering sex and gender norms (e.g., is this why caregivers may be less likely to observe ADHD‐related impacts in females than males?). Relatedly, there is also a need for investigation in possible sex differences in ADHD‐related stigma (e.g., do bias or stigma contribute to females with ADHD having a potentially higher threshold for diagnosis?; Sonuga‐Barke et al., 2023). To test these types of questions, it is important to ensure that studies are adequately powered to test for sex differences—if a sufficient number of females with ADHD are not included, questions related to sex will remain stubbornly unaddressed and underappreciated.

Well‐powered studies will also be positioned to test developmentally informed, potentially sex‐specific mechanisms and moderating factors. For instance, we recently found in a sample of early adolescents that higher thwarted belongingness (i.e., unmet need for social connection) was associated with higher depressive symptoms and suicidal ideation particularly for females with ADHD (Becker et al., 2026). Of course, just as ADHD science has disproportionately included and focused on males, so too has it disproportionately focused on risk‐related processes and negative outcomes. Studies that adopt a risk‐resilience framework should be a high priority so that our growing understanding of females with ADHD develops in tandem with growing understanding of potential strengths and resilience promoting processes (Dvorsky & Becker, 2025). For instance, although sex differences were not examined, Hendry et al. (2025) in the current issue showed that young children with ADHD traits and/or a family history of ADHD had stronger problem‐solving skills relative to peers without a family history of ADHD during a motivating, ecologically valid task. Together, the studies in this issue point to the value of methodologically rich designs that consider both risk and resilience in developmental pathways, offering a template for future studies to simultaneously consider sex differences and resilience promoting factors. To continue advancing our understanding, assessment, and treatment of females with ADHD using methodologically rigorous approaches, sustained commitment and collaborative efforts will be required.

As in any area, the execution and dissemination of rigorous research require a community—research teams, editors, reviewers, readers who apply learnings to their own research, practice, policy‐making, and advocacy, in addition to research participants and the engagement and involvement of patients with lived experience (Bellato & Seker, 2025). This is certainly the case for the research that finds its home among the pages of *JCPP Advances*. There is inherent uncertainty when launching a new journal. We reflect back with gratitude to the community that has allowed us to learn, succeed, and grow during these exciting and fruitful first 5 years. But the window ahead is larger than the rearview mirror, and we are committed and excited to build on these past 5 years as we look towards the future. Thank you for being part of the *JCPP Advances* community, and we look forward to the next 5 years ahead!

## AUTHOR CONTRIBUTIONS

**Stephen P. Becker**: Conceptualization; writing—original draft.

## CONFLICT OF INTEREST STATEMENT

During the previous 12 months, Stephen Becker reports receiving royalties for a book from Guilford Press and for continuing education courses from Premier Educational Seminars, Inc. (PESI) and receiving speaker fees from Wumbox; all outside the submitted work. Stephen Becker is a Joint Editor of *JCPP Advances*.

## FUNDING INFORMATION

National Institute of Mental Health; R01MH122415; Institute of Education Science; R324A240155; R305A240106

## ETHICAL CONSIDERATIONS

The author has nothing to report.

## Data Availability

Data sharing not applicable to this article as no datasets were generated or analyzed.
